# A novel endovascular perfusion branch strategy to reduce the risk of spinal cord ischemia in complex thoracoabdominal aortic aneurysm repair

**DOI:** 10.1016/j.jvscit.2025.101846

**Published:** 2025-05-19

**Authors:** Kelly Feng, Russell Bourchier, Andrew Holden, Anastasia Dean

**Affiliations:** a Auckland Regional Vascular Service, Auckland City Hospital, Auckland, New Zealand; b Northern Region Interventional Radiology Service, Auckland City Hospital, Auckland, New Zealand

**Keywords:** Thoracoabdominal aneurysm, Spinal cord ischemia, TBAD, TEVAR, Case report

## Abstract

We present the case of a 39-year-old woman with Turner syndrome and a 65-mm postdissection type 2 thoracoabdominal aortic aneurysm, with a coarctation and extremely narrow true lumen. The patient underwent thoracic and abdominal debranching followed by endograft placement from the ascending aorta to the infrarenal aorta through the false lumen. Self-expanding stents were deployed from the iliosplenic graft, through the dissected celiac artery, and into the aortic true lumen to reduce the risk of spinal cord ischemia. Four weeks later, after test occlusion of the perfusion branch under local anesthesia, the stent was occluded with a vascular plug.

Spinal cord ischemia (SCI) incidence remains significant after complex endovascular repair of thoracoabdominal aortic aneurysms (TAAAs) despite overall reduction in mortality and morbidity.^1^ Risk reduction strategies include optimizing postoperative blood pressure, ensuring adequate hemoglobin,^2^ staging of procedures,^3^ and prophylactic or therapeutic cerebrospinal fluid drainage (CSFD).^4^ Newer strategies include minimally invasive segmental artery coil embolization.^5^ We describe a novel strategy where self-expanding stents were deployed from the iliosplenic graft, through the dissected celiac artery (CA), into the aortic true lumen (TL) to maintain a channel to multiple intercostal arteries. After 4 weeks, this endovascular perfusion branch was occluded after a balloon occlusion test. The patient provided full publication consent.

## Case report

**Fig 1:**
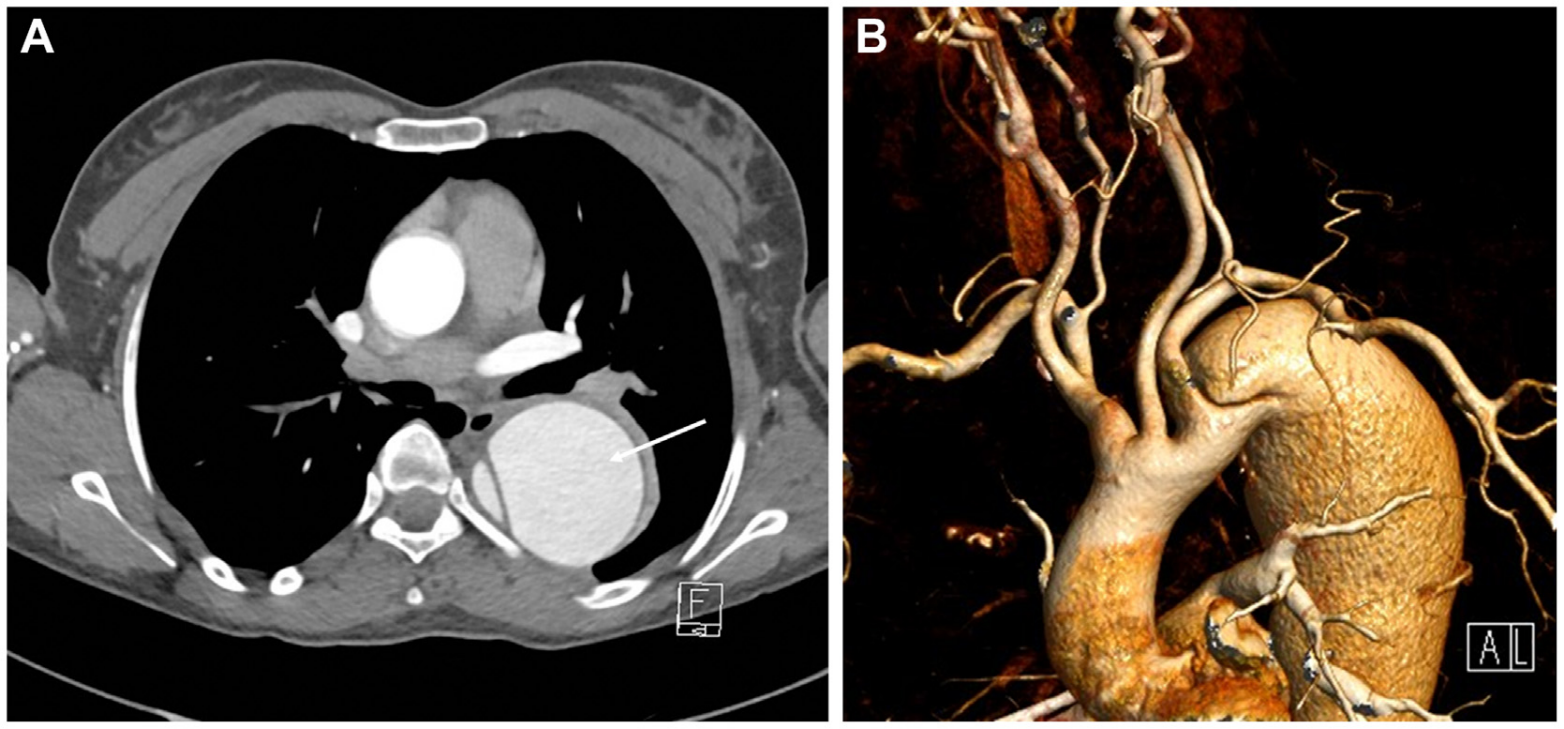
Preoperative computed tomography (CT) arteriogram. **(A)** Axial CT image of the thoracic aorta. Note the aneurysmal descending thoracic aorta composed of an aneurysmal false lumen (FL) (*arrow*) and narrowed true lumen (TL) **(B)** Volume-rendered reconstruction. Note the dissection arises immediately distal to the left subclavian artery with crowding of the arteries arising from the arch.

A 39-year-old patient with Turner syndrome presented with a 3-month history of dry cough and mediastinal widening on chest radiography. She was otherwise well without history of drug use, trauma, or pregnancy. Blood pressure was 159/70 mm Hg. Examination and blood tests were otherwise normal. Computed tomography angiography demonstrated a postdissection TAAA with maximum diameter 65 mm, extending from the left subclavian artery to the infrarenal aorta (Fig 1). An extremely constrained TL (<10 mm in the descending thoracic aorta, 18 × 6 mm in the visceral aorta) supplied the CA, superior mesenteric artery, right renal artery and inferior mesenteric artery. The aneurysmal false lumen (FL) supplied the left renal artery. The aortic arch was unfavorable with dissection-related coarctation and closely grouped supra-aortic vessels. After a multidisciplinary aortic meeting, open TAAA repair was offered given her age and connective tissue disease, which she declined. After extensive conversations, the patient agreed to a staged hybrid surgical repair, accepting the increased time to aneurysm sealing.

Initial debranching of the thoracic, abdominal and neck vessels was completed in three stages. A trifurcated Dacron graft to the innominate, left common carotid and left axillary arteries was anastomosed low onto the uniform-diameter segment of the ascending aorta (zone 0), allowing proximal extension of a thoracic endograft. Abdominal debranch involved bifurcated Gelsoft Plus Dacron grafts (14 × 7 mm, 12 × 6 mm) (Vascutek, Inchinnan, Scotland) from the distal left common iliac artery to both renal arteries, the superior mesenteric artery and the splenic artery—the CA and common hepatic artery were dissected and not appropriate for anastomosis. Erroneous ligation of the native axillary artery proximal to the initial bypass graft prevented retrograde perfusion, hence a left carotid-subclavian bypass was completed given codominant vertebral arteries.

**Fig 2:**
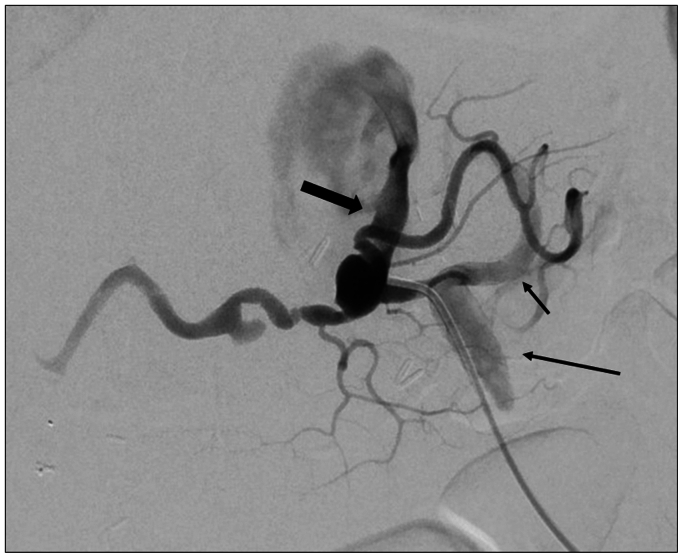
Angiography of the iliosplenic graft. Note the catheter has traversed the iliosplenic graft (*long arrow*), into the splenic artery (*short arrow*). Contrast is refluxing through the celiac artery (CA) trunk (*bold arrow*) into the true lumen (TL).

For the endovascular procedure, bilateral percutaneous groin access was obtained. A guidewire traversing the iliosplenic graft into the CA preferentially passed into the FL, so the aortic and CA TL was accessed via the right groin. The guidewire was snared in the iliosplenic graft to create a through-and-through wire for retrograde passage of a 6F × 45 cm Destination (Terumo, Tokyo, Japan) sheath to the CA ostium and buddy Rosen wire into the aortic TL (Fig 2). Everflex (Medtronic, Dublin, Ireland) self-expanding bare stents measuring 7 mm × 60 mm and 7 mm × 80 mm were placed from the iliosplenic graft through the CA into the TL, securing a channel to a number of intercostal arteries.

**Fig 3:**
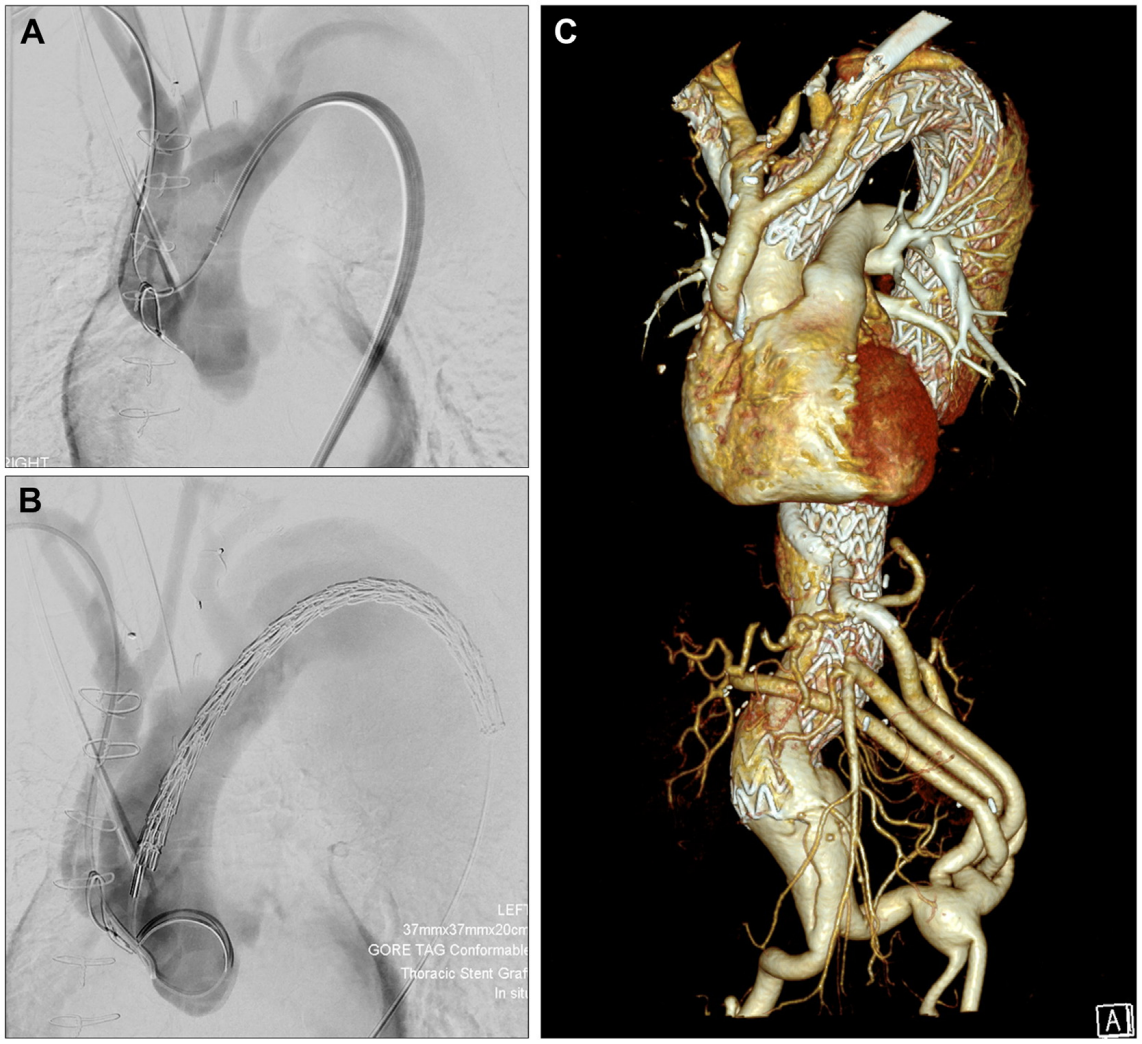
Retrograde delivery of thoracic endografts. **(A)** Guidewire is delivered retrograde from a right radial artery approach, through the debranching graft and aortic coarctation and subsequently snared in the descending thoracic aorta to provide a through-and-through guidewire. **(B)** Endograft is then delivered retrograde via a right groin access. **(C)** Volume-rendered reconstruction of the completed thoracoabdominal aortic aneurysm repair.

Retrograde guidewire passage through the coarctation was unsuccessful, so right radial access was obtained and the guidewire was snared in the distal thoracic aorta to create a through-and-through wire, facilitating retrograde passage of a sheath and buddy wire, which was exchanged for a double curve Lunderquist extra stiff wire (Cook Medical, Bloomington, IN) into the ascending aorta. The left subclavian artery was embolized with an 8-mm Amplatzer 1 plug (Abbott, Abbott Park, IL) proximal to the vertebral artery. Subsequently, four Gore TAG Conformable Thoracic Stent Grafts (W. L. Gore & Associates, Flagstaff, AZ) measuring 40 mm × 40 mm × 20 cm and 37 mm × 37 mm × 20 cm were placed from the ascending to the infrarenal aorta through the FL, which communicated freely with the iliac arteries distally (Fig 3).

**Fig 4:**
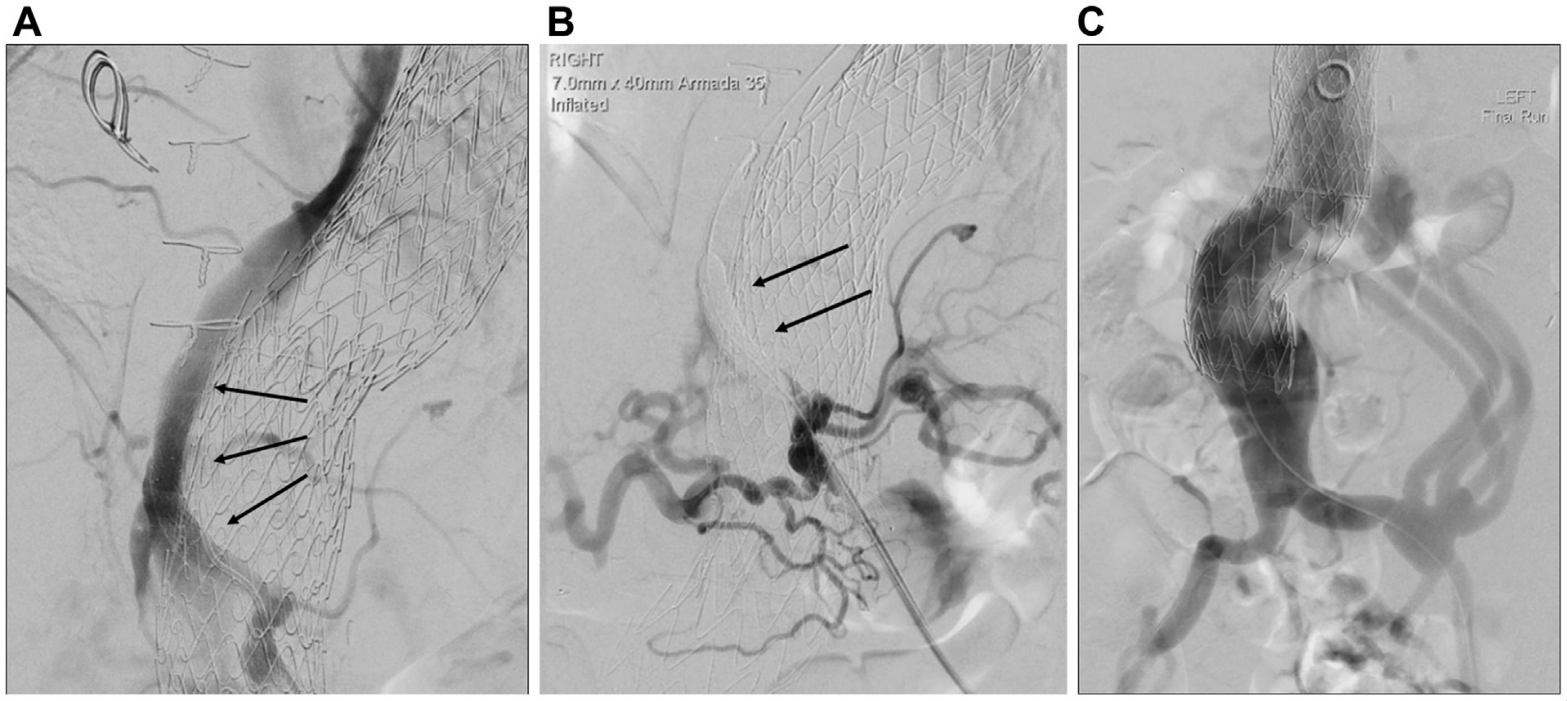
Occlusion of the endovascular perfusion branch. **(A)** Angiography via a catheter in the celiac artery (CA). Note the stented communication between the CA and aortic true lumen (TL) (*arrows*). **(B)** Temporary occlusion of the perfusion branch with an angioplasty balloon (*arrows*). The branch was subsequently occluded with a permanent vascular plug. **(C)** Angiography showing the distal end of the endograft and the patent branches of the iliovisceral graft arising from the left common iliac artery.

**Fig 5:**
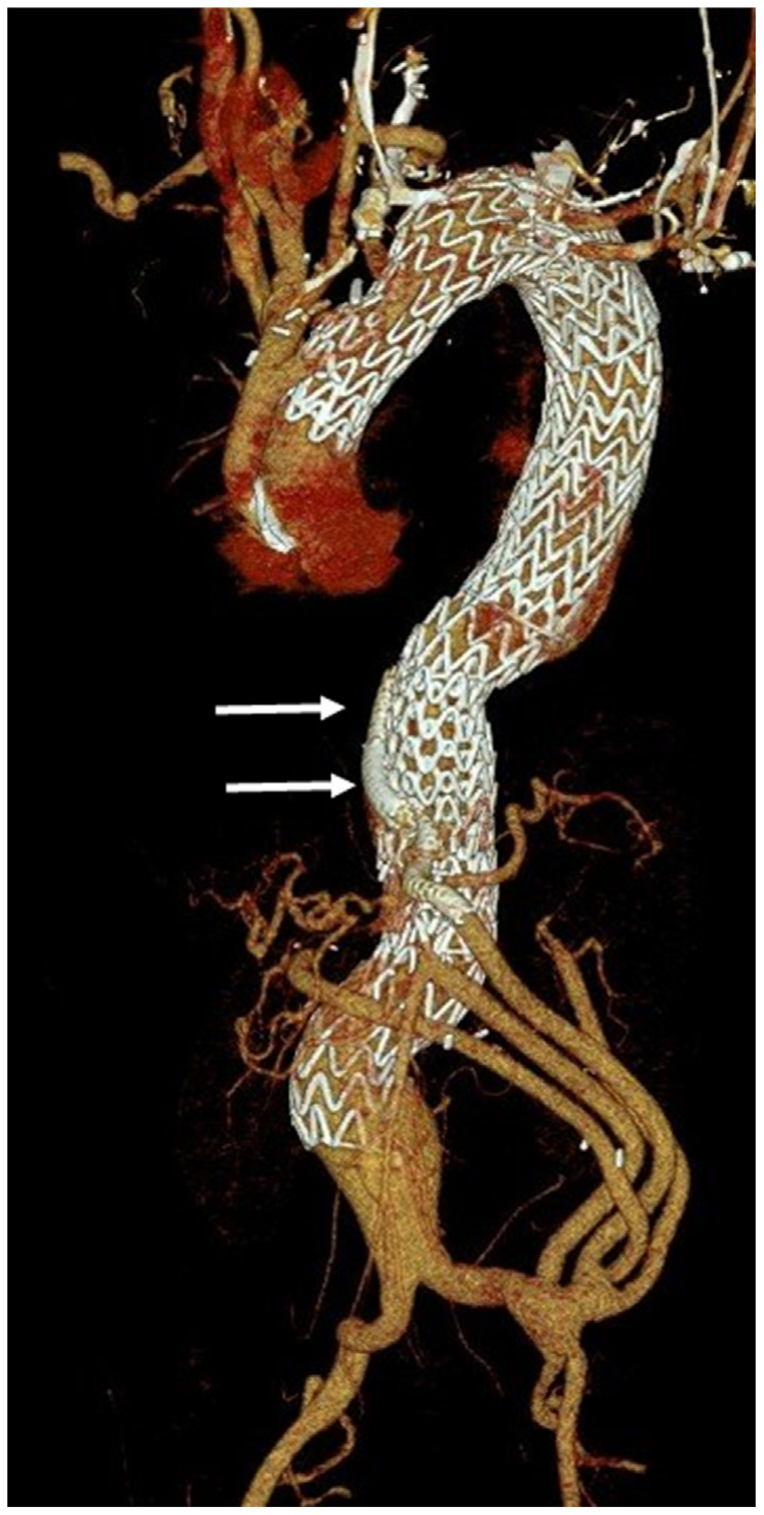
Volume-rendered reconstruction of a postoperative computed tomography (CT) arteriogram. Note the perfusion branch is occluded (*arrows*).

Postoperatively, neuromonitoring was completed in a high-dependency unit and hypotension and anemia avoided. Four weeks later, an Armada 035 PTA catheter (7 mm × 40 mm) (Abbott) occluded the endovascular perfusion branch under local anesthesia (Fig 4). After 10 minutes without sensorimotor change, the stent was occluded with an Amplatzer Vascular Plug (10 mm × 7 mm) (Abbott). Had neurological deficits developed during test occlusion, the balloon would have been deflated immediately. If symptoms failed to resolve rapidly, or occurred after plug insertion, a therapeutic CSFD would have been inserted promptly. Recovery was uneventful and the 6-month computed tomography angiography demonstrated satisfactory appearances with an occluded perfusion branch (Fig 5). The patient remains on surveillance.

## Discussion

SCI after TAAA repair may result from a reduction of spinal cord perfusion, ischemia-reperfusion mechanism with spinal cord edema and increased CSF pressure, and/or late thrombosis of intercostal arteries.^6^ Coverage of intercostal arteries and risk of embolization and local dissection intrinsically contributes to SCI risk with endovascular repair—approximately 3.9% (compared with 5.7% in open repair) up to 13.4% in Crawford extent II repair.^7^ Known risk factors include extent of the aneurysm, length of aortic coverage required,^1^ and female sex,^8^ which placed this patient in the highest risk group for developing SCI. The extensive aortic involvement, severely narrow TL and dissected target arteries—challenges distinctive of postdissection aneurysms—with a lack of suitable area for temporary sealing to facilitate staged aortic coverage required consideration of nonstandard SCI prevention techniques.

Preconditioning spinal cord vasculature to better tolerate occlusion of supplying vessels is one concept behind staging, temporary aneurysm sac perfusion^9^ and minimally invasive segmental artery coil embolization^10^—another promising option to consider in such a diffusely dilated aorta. Our dedicated channel to an island of intercostals arising from the TL between T8 and T12, the region of the artery of Adamkiewicz, theoretically reduces the decrease in spinal cord perfusion and encourages collateral network development. We also maintained perfusion to important collaterals—the left subclavian artery and hypogastric arteries.

Balloon occlusion of the perfusion branch under local anesthesia before plug insertion allowed observation of sensorimotor function and avoidance of prophylactic CSFD. No clear guidelines exist for optimal balloon occlusion time, and more research is required.

We acknowledge that current guidelines do not recommend visceral debranching in combination with endovascular aneurysm endograft as first-line treatment for complex TAAAs, and time to aneurysm sealing was increased. Complex endovascular options including off-the-shelf and custom-made device endografts were considered but not selected given the TL was less than one-half of the minimum diameter suggested for off-the-shelf inner branch aortic endografts. Cannulation and stenting of target vessels for placement of branched endografts through the FL would have been challenging even with advanced techniques. Successful bridging carried substantial risk of instability given tortuosity and length of bridging stent potentially required. Recent results with inner branch technology emphasized the potential reduced durability, especially in renal artery bridging stents.^11^ Electrified wire septotomy is a plausible technique to facilitate endograft placement,^12^ but is not established universally and carries the risk of serious complications, particularly in this patient with connective tissue disease.

Complex anatomical and patient factors commonly limit postdissection TAAA repair options and SCI risk remains significant. We demonstrate a novel endovascular strategy that potentially reduces SCI risk in this setting. Further research is required to establish optimal timing for staging and duration of balloon occlusion.

## Funding

None.

## Disclosures

None.
